# Stress as a Common Integrative Measure Between Biology and Engineering in Bone Healing and Remodeling

**DOI:** 10.3390/bioengineering13070758

**Published:** 2026-06-29

**Authors:** Nenad Šešić, Marijo Bekić, Maro Jelić, Miho Klaić, Antun Bekić, Cecilija Rotim, Petra Bagavac

**Affiliations:** 1 Dubrovnik County Hospital, 20000 Dubrovnik, Croatia; nenad.sesic@yahoo.com; 2 Maritime Department, University of Dubrovnik, 20000 Dubrovnik, Croatia; maro.jelic@uni-du.hr (M.J.); miho.klaic@uni-du.hr (M.K.); 3 School of Medicine, Catholic University of Croatia, 10000 Zagreb, Croatia; bekicantun@gmail.com; 4 Faculty of Dental Medicine and Health, University of Osijek, 31000 Osijek, Croatia; crotim@rotim.hr; 5 Faculty of Electrical Engineering, Mechanical Engineering and Naval Architecture, University of Split, 21000 Split, Croatia; pstrizak@fesb.hr

**Keywords:** stress, bone healing, bone remodeling, callus formation, finite element method (FEM), stress distribution, non-union, biomechanical adaptation

## Abstract

This study proposes mechanical stress as a common integrative parameter that connects biological bone adaptation with engineering mechanics. In engineering, stress is defined as the internal reactive force per unit area within a loaded body; in clinical practice, its biological consequences can be observed on radiographs, CT, and MRI as callus formation, hypertrophy, or oedema, and perceived by the patient as localised pain. Three-dimensional geometry models were constructed in Autodesk Fusion and Inventor, and stress distributions were visualised using Abaqus finite element analysis. Representative clinical cases (*n* = 5) were drawn from the radiographic archive of Dubrovnik County Hospital. The analysis showed that regions of elevated stress predicted by finite element models corresponded spatially with areas of callus growth observed on clinical radiographs, and that the presence or absence of stress correlated with subjective pain reports. These findings suggest that stress may serve as a clinically and biomechanically relevant parameter bridging radiographic observation and engineering analysis of bone healing and remodelling.

## 1. Introduction

Bone is a hierarchically organised biological material whose mechanical behaviour depends not only on mineral density but also on microstructural organisation, porosity, anisotropy, and the spatial distribution of its constituent phases. Although densitometric parameters are widely used to assess bone quality, they do not fully account for differences in stiffness, strength, or fracture susceptibility. This limitation has motivated the search for additional descriptors that capture structural complexity and relate it to mechanical performance [1,2,3].

Among such descriptors, fractal dimension (FD) has received increasing attention as a quantitative, non-dimensional measure of bone microstructure and density distribution. Sánchez-Molina et al. reported a clear correlation between FD derived from micro-CT images and experimentally measured Young’s modulus and failure stress in human cortical rib bone [1]. Velázquez-Ameijide et al. extended these findings to a larger set of post-mortem specimens, confirming significant associations between FD and tensile strength, yield stress, and work to fracture [2]. García-Vilana et al. further showed that FD increased as cortical bone density decreased and that it captured material heterogeneity, including water content, thereby providing a more direct physical interpretation of this parameter [3]. In osteoporotic individuals, Arnold et al. showed that combining μCT-derived FD with finite element analysis improved fracture prediction beyond densitometric assessment alone [4].

The clinical relevance of image-based structural analysis extends beyond cortical bone. Yeni et al. showed that cancellous bone textural properties, together with density parameters, predicted vertebral wedge strength [5]. Hirvasniemi et al. confirmed that density- and structure-related parameters from plain radiographs correlated with three-dimensional microarchitecture [6], while Roberts et al. demonstrated that image texture measures from dental panoramic radiographs contributed to osteoporosis identification [7]. Texture-based analysis has also been applied to monitor periprosthetic bone adaptation and osteoarthritis progression [8,9].

While image-derived descriptors provide valuable structural information, predictive modelling of bone behaviour also requires constitutive formulations capable of describing anisotropy, post-yield deformation, and damage. Schwiedrzik and Zysset proposed an anisotropic elastic–viscoplastic damage model that captures both elastic and post-yield behaviour [10], and Hamandi and co-workers showed that anisotropic, CT-based material descriptions provide a more realistic representation of bone behaviour than simplified elastic models [11,12]. These studies suggest that structural descriptors such as FD may be most informative when integrated with mechanically grounded finite element approaches.

Taken together, the available literature indicates that bone mechanical competence cannot be explained by density alone. Fractal dimension provides additional information on structural complexity, and several studies have demonstrated significant associations between FD and mechanical variables. However, the mechanistic relationship among density distribution, tissue composition, and local failure remains incompletely understood. This provides a rationale for integrating structural image analysis with mechanical testing and numerical modelling [1,2,3,4,10,11,12].

Trabecular bone plays a central role in skeletal mechanical competence by continuously adapting its architecture to physiological demands. Smotrova-Kayali et al. demonstrated that an optimization-based finite element bone-adaptation model could reproduce in vivo trabecular remodelling in the human distal tibia with high element-by-element agreement [13]. The structural importance of trabecular bone is further highlighted by Jia et al., who reported that removal of cancellous bone reduced proximal femoral strength by 67.2% in lateral-fall simulations, with failure consistently initiating at the endosteal–trabecular junction [14].

At the tissue level, Huang et al. showed that corrected tissue modulus, strength, and effective modulus followed significant power-law relationships with bone volume fraction in progressively attenuated trabecular samples [15]. From a fracture-mechanics perspective, Hudyma et al. provided proof-of-concept evidence that finite-discrete element modelling can qualitatively reproduce cortical bone fracture propagation under compressive loading [16]. The clinical relevance of trabecular bone quality was further emphasized by Ye et al., who linked lower proximal femoral trabecular CT values with reduced stability after PFNA fixation, showing that impaired trabecular bone quality shifts load to the implant–bone construct [17].

The relationship between mechanical loading and fracture healing has been extensively studied at both experimental and computational levels. Claes and Heigele demonstrated that local magnitudes of stress and strain along bony surfaces predict the course of tissue differentiation at the fracture site [18]. Perren’s interfragmentary strain theory established that different tissues tolerate different strain magnitudes, providing a biomechanical rationale for why some fractures heal with callus while others progress to non-union [19]. Einhorn and Gerstenfeld reviewed the complex interplay between mechanical, vascular, and cellular mechanisms governing fracture repair [20]. Despite these advances, a unified clinical-level framework that allows practitioners to interpret visible radiographic features as direct consequences of internal stress distribution remains lacking. The present study addresses this gap by proposing stress as a common integrative measure between biological observation and engineering analysis.

## 2. Materials and Methods

### 2.1. Study Design

**Figure 1:**
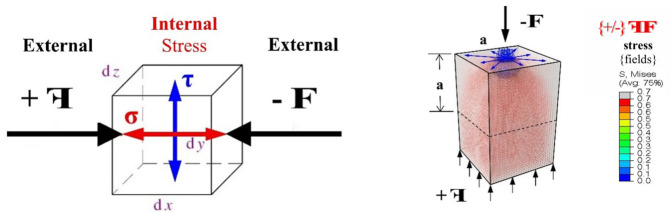
Schematic representation of the internal stress state within a three-dimensional body subjected to external loading. (**Left**) Conceptual diagram illustrating the normal stress (σ) and shear stress (τ) components acting on an infinitesimal element extracted from the loaded body. (**Right**) Corresponding finite element method (FEM) visualisation of the von Mises stress field distribution under the same loading configuration, demonstrating how internal stresses redistribute through the volume. The colour gradient represents relative stress magnitude, with warmer colours indicating higher stress concentrations.

This study was designed as a conceptual biomechanical analysis that explores stress as a common integrative measure linking biological bone adaptation and engineering mechanics. No new mechanical experiments were performed. Instead, the study combined three complementary approaches: (i) theoretical interpretation of stress as an internal response to external loading (Figure 1); (ii) qualitative analysis of clinical radiographic examples of bone healing, non-union, and degeneration; and (iii) illustrative finite element visualisation of stress-field distribution in simplified geometries with localised loading and structural discontinuities. The objective was to examine whether visible biological form can be interpreted as a consequence of local stress distribution and whether such an interpretation can improve communication between medical and engineering perspectives.

#### Clinical Case Selection

Representative clinical cases were selected retrospectively from the radiographic archive of Dubrovnik County Hospital, covering surgically and conservatively treated patients seen between 2015 and 2025. Five patients across four biomechanical categories were chosen to illustrate different mechanical situations: (a) paediatric forearm and humeral fractures treated conservatively and surgically, showing asymmetric callus formation and complete remodelling (*n* = 2 patients, age 4 and 9 years); (b) an adult open tibial–fibular fracture treated with external fixation, illustrating prolonged remodelling with residual asymmetry (*n* = 1 patient, male, age 46); (c) a humeral supracondylar non-union demonstrating shear-dominant mechanical failure (*n* = 1 patient); and (d) a degenerative joint condition interpreted as decompensated stress balance (*n* = 1 patient). Inclusion criteria were: availability of sequential radiographic follow-up, clearly identifiable callus or remodelling patterns, and clinical documentation of treatment and outcome. Cases were selected to represent mechanically distinct categories rather than to constitute a statistical sample. All images were fully anonymised prior to analysis.

### 2.2. Conceptual Biomechanical Framework

The central premise of this study is that external forces generate an internal stress field composed of normal and shear components, and that bone adaptation can be interpreted as a time-dependent structural response to that field. In engineering terms, stress is the internal reactive force per unit area within a loaded body. In the biological interpretation adopted here, stress additionally acts as a form-generating stimulus whose consequences are expressed through visible structural adaptation—callus formation, remodelling, and, under adverse mechanical conditions, non-union or degenerative change. This framework therefore treats bone morphology as a mechanically conditioned response rather than a purely static anatomical feature.

### 2.3. Illustrative Case Categories

Two mechanically distinct situations were considered.

**Figure 2:**
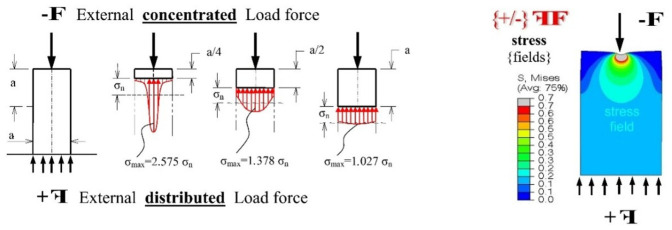
Two mechanically distinct stress situations relevant to bone fracture interpretation. (**Left**) Concentrated external load applied to a body, with stress redistribution following Saint-Venant’s principle: the diagram shows decreasing peak stress ratios (σmax) at progressively greater distances (a/4, a/2, a) from the point of load application, illustrating how localised forces dissipate through the structure. (**Right**) Corresponding FEM visualisation (von Mises stress) of the same concentrated loading scenario, confirming the spatial decay of stress concentration predicted by Saint-Venant’s principle. This case underpins the interpretation of asymmetric callus formation near fracture sites subjected to localised loading.

In Case 1, the analysis addressed concentrated loading, where the local stress field redistributes with distance from the site of force application, following Saint-Venant-type reasoning (Figure 2, left). This case was used to interpret asymmetric callus development during early fracture healing. According to the conceptual model, where local stress is present, a biologically adaptive reaction may develop in the form of callus, with the spatial distribution of newly formed tissue reflecting the underlying stress imbalance.

**Figure 3:**
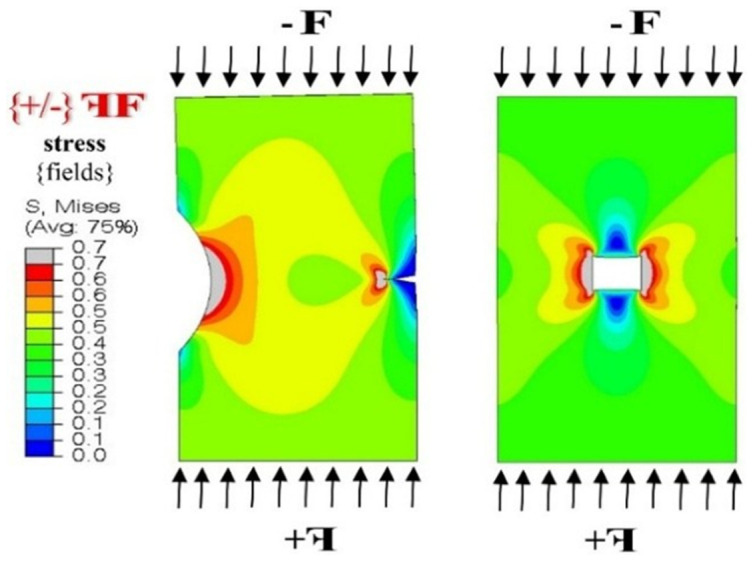
Finite element visualisation of stress concentration near geometric discontinuities under distributed external loading. The von Mises stress colour map reveals intensified stress fields in restricted regions immediately adjacent to the discontinuity (hole/defect), with symmetric stress patterns corresponding to the orientation of the applied compressive load. Regions of elevated stress (warmer colours) surround the discontinuity, while stress diminishes at greater distances. This configuration models the biomechanical scenario at a fracture site or bone defect, where geometric interruption of load-bearing cross-section produces local stress amplification that may drive adaptive biological responses such as callus formation or remodelling.

In Case 2, the analysis addressed stress concentration near geometrical discontinuities, such as notches, holes, defects, fracture surfaces, or abrupt shape changes (Figure 3). These situations are mechanically important because discontinuities alter local force transfer and intensify stress in restricted regions. Within the present framework, such stress concentrations were interpreted as potential drivers of local bone remodelling, structural compensation, or failed healing when the mechanical environment becomes unfavourable.

#### FINITE Element Visualisation

**Figure 4:**
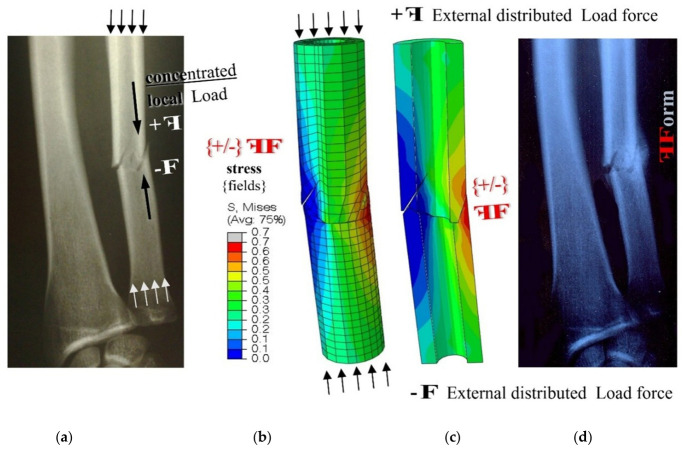
Integration of clinical radiographic and computational finite element data, illustrating the correspondence between observed biological response and predicted stress distribution. From left to right: (**a**) radiograph of a long bone fracture with concentrated local loading and visible asymmetric callus formation; (**b**) FEM mesh of a simplified cylindrical model with a geometric discontinuity under distributed compressive loading; (**c**) von Mises stress distribution showing stress concentration at the discontinuity site, with the colour gradient indicating relative stress magnitude; (**d**) clinical radiograph demonstrating the biological adaptive response (Form) to the local stress field, where callus geometry corresponds spatially to the region of elevated mechanical stress. Arrows on the clinical radiographs indicate the direction of applied mechanical loading: downward arrows repre-sent concentrated compressive force and upward arrows represent the distributed reactive force. This sequence represents the core methodology of the present study: linking visible bone form to the underlying stress environment.

Finite element visualisation was used as an illustrative tool to examine the relative distribution of three-dimensional stress fields near structural discontinuities. Three-dimensional geometry models were designed in Autodesk Fusion and Inventor, and stress analysis was performed in Abaqus FEM (Figure 4). A nominal external force was applied in order to observe relative stress redistribution rather than to derive specimen-specific absolute values. Accordingly, the finite element component of this work was interpreted qualitatively, with emphasis placed on the spatial pattern of stress concentration and its possible correspondence to visible biological adaptation.

### 2.4. Finite Element Model Parameters

The geometry consisted of simplified cylindrical and prismatic bodies with intentional geometric discontinuities (holes, notches, and transverse cuts) designed to represent idealized fracture-site and remodelling-zone configurations. Models were meshed using second-order tetrahedral elements (C3D10) with a global element size of 1.0 mm, refined to 0.3 mm in the vicinity of discontinuities. Mesh convergence was verified by comparing von Mises stress maxima across three successive refinements, confirming less than 3% variation at the finest level. Material properties were assigned as homogeneous, isotropic, and linearly elastic (Young’s modulus E = 17 GPa, Poisson’s ratio ν = 0.3), representing average cortical bone values from the literature [10,11]. Boundary conditions consisted of fixed displacement at one end and a distributed nominal compressive force at the opposite end. Von Mises equivalent stress was selected as the output variable for visualisation because it provides a single scalar representation of the multiaxial stress state, enabling intuitive visual comparison with clinical observations. It is acknowledged that von Mises stress is a scalar invariant derived from the deviatoric stress tensor and does not distinguish between tensile and compressive stress components; the implications of this choice are discussed in Section 4.6. Because the purpose of the FEM component was illustrative rather than patient-specific, no attempt was made to replicate individual anatomical geometry or heterogeneous tissue properties.

#### Clinical and Radiographic Interpretation

**Figure 5:**
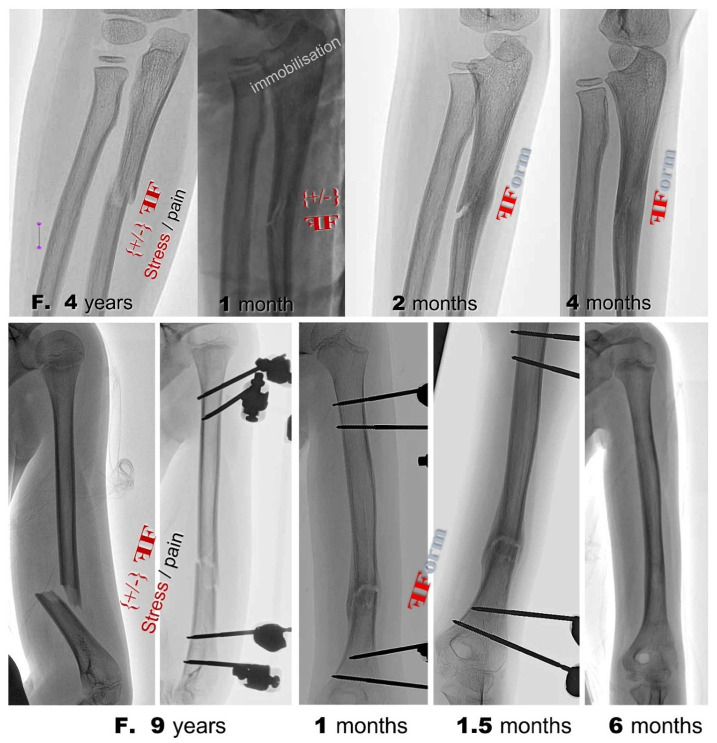
Sequential radiographic documentation of fracture healing in two paediatric patients, illustrating the time course of stress-driven bone adaptation. (**Top row**) Forearm fracture in a 4-year-old child treated conservatively with immobilisation: asymmetric callus formation is visible at 1 month post-injury, progressive remodelling with partial cortical restoration at 2 months, and near-complete structural reconstitution at 4 months. (**Bottom row**) Humeral fracture in a 9-year-old child treated surgically with percutaneous external fixation: initial callus formation at 1 month, continued biological adaptation at 1.5 months, and complete bone remodelling with restored cortical continuity at 6 months. Stress (σ) and pain indicators are annotated on the acute-phase images. The rapid and complete remodelling observed in both paediatric cases is consistent with the high biological regenerative capacity in children, where the mechanical stimulus is efficiently transduced into adaptive bone formation.

**Figure 6:**
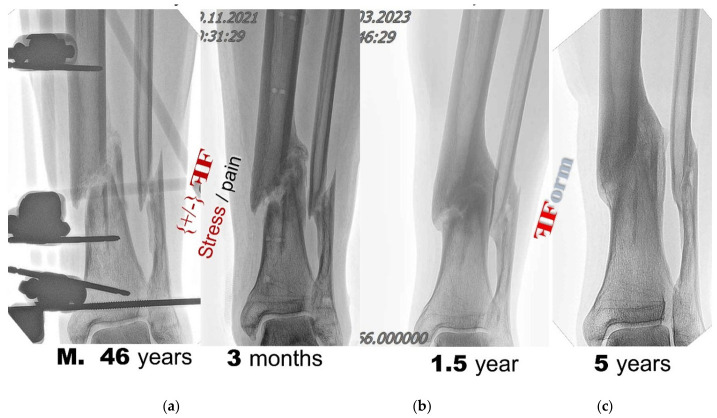
Open fracture of the tibia and fibula in a 46-year-old male patient, treated surgically with percutaneous external fixation. Sequential radiographic follow-up documents the long-term healing trajectory: (**a**) at 3 months post-injury, with the external fixation device in situ, asymmetric callus formation is evident at the fracture site (stress and pain indicators annotated); (**b**) at 1.5 years, progressive bone remodelling with partial cortical bridging; (**c**) at 5 years, continued structural adaptation with functional restoration, although complete anatomical remodelling remains incomplete. Unlike the paediatric cases (Figure 5), adult bone remodelling proceeds over a substantially longer time course. The case illustrates how the mechanical stress environment drives callus formation and progressive adaptation, while biological factors (age, vascular supply, fracture severity) modulate the rate and completeness of the remodelling process.

**Figure 7:**
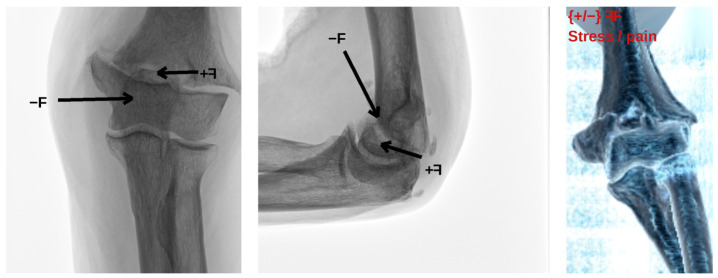
Humeral supracondylar non-union demonstrating a mechanically decompensated healing environment. (**Left**,**centre**) Anteroposterior and lateral radiographic views revealing persistent fracture discontinuity with no evidence of bridging callus. (**Right**) Three-dimensional CT reconstruction confirming the non-united fracture gap. In this case, the predominant loading direction acts transversely to the fracture surface, producing shear stress that exceeds the stabilising effect of normal compressive stress. Within the proposed conceptual framework, this unfavourable ratio of shear-to-normal stress creates a mechanical environment that inhibits stable callus bridging and consolidation, in contrast to the successfully healing fractures presented in Figure 5 and Figure 6 where compressive stress dominated.

**Figure 8:**
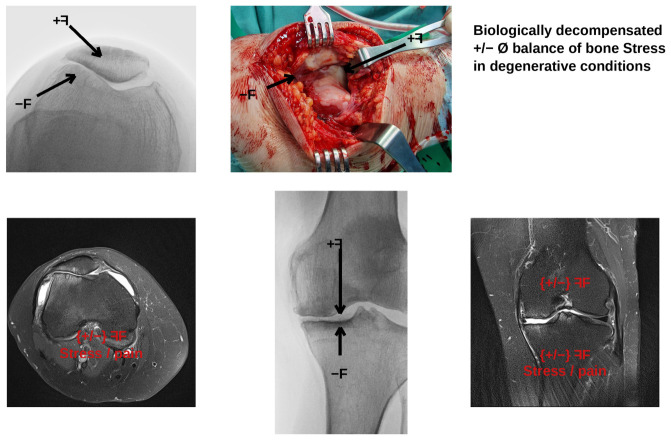
Degenerative joint condition interpreted within the proposed framework as a biologically decompensated stress balance. (**Top**) Intraoperative photograph of the articular surface showing cartilage degeneration and subchondral exposure, with superimposed force vectors indicating the local stress environment at the joint interface. (**Bottom**) Multimodal imaging of the knee joint: MRI in coronal and sagittal planes alongside a weight-bearing radiograph, demonstrating progressive structural deterioration in regions subjected to persistent stress imbalance. Stress (σ) and pain annotations indicate the spatial correspondence between areas of chronic mechanical overloading and tissue decompensation. This case extends the conceptual framework beyond acute fracture healing to chronic degenerative conditions, where prolonged unfavourable stress distribution contributes to progressive joint deterioration rather than adaptive remodelling.

The conceptual framework was further examined using representative radiographic examples: asymmetric callus formation in healing fractures in paediatric and adult patients (Figure 5 and Figure 6), humeral supracondylar non-union (Figure 7), and degenerative joint conditions (Figure 8). These examples were interpreted biomechanically by relating visible tissue response to the local balance between normal and shear stress. Particular attention was paid to the situation in which dominant shear stress acted transversely to the fracture surface, as this was considered mechanically unfavourable for union. Degenerative conditions were also included as examples of biologically decompensated stress balance.

## 3. Results

### 3.1. Stress as a Visible Structural Measure

The conceptual analysis indicates that stress can be interpreted not only as a scalar or tensorial mechanical variable but also as a visible structural measure expressed through bone form. Within the adopted framework, local bone architecture, callus geometry, and remodelling patterns represent manifestations of the internal stress field generated by external loading. This interpretation supports the use of stress as a shared explanatory variable that links biological observation with engineering analysis.

### 3.2. Case 1: Localized Loading and Asymmetric Callus Formation

In the first case category, concentrated external loading produced a non-uniform stress distribution that was reflected biologically by asymmetric callus formation during the acute healing phase (Figure 5 and Figure 6). The presence of local stress corresponded with biological adaptation expressed as bone callus. Radiographic follow-up showed that new tissue did not form symmetrically around the fracture site but preferentially developed on the mechanically dominant side of the stress field. In the paediatric patients (Figure 5), complete remodelling was achieved within 4–6 months, whereas in the adult patient (Figure 6) remodelling continued over several years. These observations support the view that the spatial distribution of early repair tissue is governed by the local stress environment.

### 3.3. Case 2: Stress Concentration Near Discontinuities and Remodelling

In the second case category, finite element visualisation confirmed that stress became concentrated near geometric discontinuities (Figure 3 and Figure 4). The biological correlate of this mechanical finding is local remodelling or compensatory adaptation of bone form: where stress concentration develops near notches, holes, or fracture-related irregularities, the corresponding biological response is structural modification aimed at restoring mechanical equilibrium. The comparison between FEM stress maps and clinical radiographs in Figure 4 illustrates this correspondence directly, reinforcing the view that bone form can be read as a consequence of sustained local stress concentration.

### 3.4. Non-Union as a Consequence of Unfavourable Stress Balance

The non-union case further refined this interpretation. In the humeral supracondylar non-union (Figure 7), the predominant loading direction was transverse to the fracture surface, producing shear stress that exceeded normal compressive stress. Within the conceptual model, this imbalance creates mechanical conditions unfavourable for union—a state of biologically decompensated healing in which the stress environment does not support stable bridging and consolidation.

### 3.5. Stress Imbalance in Degenerative Conditions

The framework was also applied to degenerative bone and joint conditions (Figure 8), which represent examples of decompensated stress balance. In these cases the biological structure no longer compensates adequately for local mechanical demand, and the radiographic manifestation of degeneration can be understood as a consequence of persistent, unresolved stress imbalance.

## 4. Discussion

### 4.1. Novelty and Relationship to Existing Frameworks

The present work proposes that stress can serve as a central common measure between biology and engineering in the interpretation of bone behaviour. In engineering, stress is typically defined as an internal response to external loading and analysed numerically or analytically. In biology, however, the same internal mechanical state may be observed indirectly through visible adaptive outcomes—callus formation, remodelling, non-union, or degeneration. The principal contribution of this study is therefore conceptual: it reframes stress from a purely numerical quantity into a biologically interpretable structural signal.

It is important to distinguish the present framework from established mechanobiology theories. Wolff’s law [21] describes a general principle that bone adapts its architecture to habitual loading, but does not specify the local mechanical variable responsible for the observed structural change. Frost’s mechanostat model [22] introduces strain thresholds (minimum effective strain) that trigger remodelling, modelling, or disuse atrophy, providing a homeostatic control framework. Pauwels’ theory [23] distinguishes between hydrostatic and deviatoric stress components as determinants of chondrogenesis versus osteogenesis, a concept later quantified by Claes and Heigele [18], who demonstrated that local magnitudes of stress and strain along bony surfaces predict the course and type of fracture healing. The interfragmentary strain theory advanced by Perren [19] further established that tissue differentiation at the fracture site is governed by the mechanical strain environment, with different tissues tolerating different strain magnitudes. More recent computational mechanoregulation approaches, such as those by Lacroix and Prendergast [24], use octahedral shear strain and interstitial fluid velocity to predict tissue differentiation at the fracture callus level. Ghiasi et al. [25] provided a comprehensive review of these mechanobiological modelling principles, while Ren and Dailey [26] extended mechanoregulation modelling to realistic three-dimensional fracture geometries with clinically relevant loading conditions. The present study does not replace or contradict these frameworks. Rather, it proposes stress as a unifying observational parameter that bridges engineering computation and clinical radiographic interpretation. While mechanoregulation models operate at the computational tissue-differentiation level, the present framework operates at the clinical–conceptual level, offering clinicians a way to interpret visible bone form as a manifestation of the internal stress field without requiring access to patient-specific computational models.

It is acknowledged that the relationship between mechanical loading and bone adaptation has been extensively studied since Wolff’s original formulation in 1892 [21]. However, the existing literature has predominantly pursued this relationship through two avenues: (a) computational mechanoregulation models that predict tissue differentiation from strain-based stimuli at the cellular and tissue scale [24,25,26], and (b) experimental studies that apply controlled loading and measure resulting bone formation or resorption [27,28]. The present work does not claim to add new experimental data or a new predictive algorithm. Instead, it offers a third, complementary perspective: a clinical–conceptual framework in which stress serves as a shared observational parameter enabling clinicians to interpret routine radiographic findings in biomechanical terms. Whereas mechanoregulation models require patient-specific geometry, material properties, and loading data that are rarely available in everyday clinical practice, the proposed framework operates with standard clinical imaging and qualitative reasoning about the local stress environment. The novelty therefore lies not in the mechanical principles themselves, which are well established, but in their systematic application as a clinical interpretive tool that bridges the communication gap between engineering analysis and orthopaedic practice.

### 4.2. Interpretation of Findings

This interpretation is consistent with the broader biomechanical understanding that bone is anisotropic, non-homogeneous, and structurally adapted to its mechanical environment. Rather than treating bone form as fixed anatomy, the present framework regards it as an optimised response to external loading over time. This perspective is particularly relevant to fracture healing, where local differences in force transfer may explain why callus develops asymmetrically and why some fractures progress towards union while others proceed towards delayed union or non-union. The illustrative case of humeral supracondylar non-union supports this view, suggesting that excessive local shear relative to normal stress may prevent stable healing.

The finite element illustrations presented here were not intended to provide patient-specific predictions but to clarify the relationship between mechanical cause and biological effect. By applying a nominal force and observing the resulting relative stress distribution, the models highlight where biologically meaningful adaptation would be expected to occur. In this way, FEM serves not only as a computational tool but also as a conceptual bridge between invisible internal mechanics and visible biological morphology.

An important methodological caveat concerns the nature of the computed stress values. Stress is not a directly measurable quantity in living bone; it can only be inferred indirectly through deformation, strain measurement, or numerical simulation. The FEM results presented in this study were obtained using simplified geometry, homogeneous isotropic material properties, and nominal loading conditions that do not reproduce the complex heterogeneous, anisotropic, and viscoelastic behaviour of real bone tissue. Consequently, the computed stress fields represent relative spatial patterns rather than absolute magnitudes. The value of these simplified models lies not in their quantitative accuracy but in their ability to reveal qualitative stress redistribution patterns—such as concentration near discontinuities and decay with distance from load application—that are consistent with well-established principles of solid mechanics and that correspond to observable biological responses in the clinical cases presented. The present study therefore uses FEM as a conceptual visualisation tool, not as a patient-specific predictive instrument.

### 4.3. Role of Biological Factors

It must be emphasized that bone healing and remodelling are inherently multifactorial processes. As reviewed by Claes et al. [29] and Einhorn and Gerstenfeld [20], the fracture repair cascade proceeds through inflammatory, reparative, and remodelling phases, each regulated by an interplay of mechanical, vascular, and biological signals. Vascularization is a prerequisite for osteogenesis; without adequate blood supply, even a mechanically favourable stress environment cannot support union. Ma et al. [30] demonstrated that mechanical loading directly influences vascularization during bone healing, with appropriate loading promoting angiogenic responses that support callus maturation and regeneration. Cellular activity, including osteoblast recruitment, osteoclast-mediated resorption, and mesenchymal stem cell differentiation, constitutes the biological effector mechanism through which the mechanical stimulus is translated into structural adaptation. Metabolic factors such as calcium and vitamin D homeostasis, hormonal regulation, age, nutrition, and systemic disease further modulate the healing response [20,29]. The present framework does not claim that stress is the sole determinant of bone behaviour. Rather, it positions stress as the shared mechanical variable that is accessible to both engineering analysis and clinical radiographic observation, while acknowledging that the biological response to any given stress field is modulated by vascular, cellular, and metabolic conditions. In this sense, stress functions as a necessary but not sufficient condition for adaptive bone formation.

### 4.4. Clinical Implications and Interdisciplinary Workflow

The study has implications for clinical biomechanics and implant design. If visible bone structure can be interpreted as the product of accumulated stress history, then radiographic and tomographic patterns may be used not merely descriptively but as indicators of the underlying mechanical state. Such a viewpoint could improve interdisciplinary communication by enabling clinicians and engineers to discuss healing, remodelling, or degeneration within a shared framework centred on stress distribution and structural response.

A practical interdisciplinary workflow emerging from this framework could proceed as follows: (1) the clinician identifies a radiographic pattern of interest, such as asymmetric callus, delayed union, or localised resorption; (2) the pattern is interpreted in terms of the expected local stress environment, distinguishing compressive-dominant from shear-dominant conditions; (3) if computational confirmation is desired, simplified FEM visualisation is used to verify whether the observed biological pattern is consistent with the predicted stress distribution; and (4) the combined clinical–mechanical interpretation informs treatment planning, including fixation strategy, load-bearing protocol, or revision assessment. This workflow does not require patient-specific modelling at every stage, but provides a structured conceptual path for translating radiographic observation into mechanically informed clinical reasoning.

### 4.5. Towards Quantitative Validation

A key limitation of the present study is its qualitative nature. To move beyond conceptual demonstration, future work should pursue semi-quantitative and quantitative validation. Possible approaches include: (a) region-of-interest mapping, in which callus area or density from sequential radiographs is measured in defined anatomical zones and correlated with FEM-predicted stress magnitudes in corresponding regions; (b) scoring-based comparison, where clinical outcomes (union, delayed union, non-union) are scored against mechanical parameters extracted from patient-specific or parametric FEM models; and (c) prospective cohort studies in which radiographic callus distribution is compared with pre-operative or intra-operative biomechanical assessment. Recent experimental work provides encouragement for such quantitative approaches: Wehrle et al. [27] demonstrated that individualized cyclic mechanical loading, guided by animal-specific micro-finite element analysis, significantly improved callus properties during the remodelling phase of fracture healing, confirming that targeted mechanical intervention based on computed strain distributions can measurably alter healing outcomes. Similarly, Branecka et al. [28] proposed a continuum remodelling model in which bone independently adapts its bulk and shear moduli in response to hydrostatic and deviatoric strain stimuli, offering a computational framework for relating local stress states to measurable structural changes. Such studies would transform the present conceptual framework into a testable hypothesis with quantifiable predictive value.

### 4.6. Limitations

Several limitations should be acknowledged. First, this is a conceptual and illustrative study, not a controlled experimental or patient-specific computational investigation; the clinical cases were selected to represent mechanically distinct categories and do not constitute a statistical sample. Second, all results are qualitative: no new absolute mechanical values, statistical analyses, or validation experiments were generated, and the correspondence between FEM stress maps and radiographic observations relies on visual interpretation, which introduces potential observer bias. Third, the FEM models employed simplified, homogeneous, isotropic material properties rather than patient-specific anisotropic tissue data, limiting their anatomical fidelity. Fourth, biological healing depends on vascular, cellular, metabolic, and temporal factors in addition to local mechanics (Section 4.3). Stress should therefore be understood not as the sole determinant of healing outcome but as a unifying biomechanical measure that helps organise biological and engineering observations within a common language.

Fifth, the use of von Mises equivalent stress as the sole output variable represents a simplification. Von Mises stress is a scalar quantity derived from the deviatoric stress tensor that combines all stress components into a single value representing distortion energy. As such, it does not distinguish between tensile and compressive stress states, which are known to exert different effects on bone remodelling: compressive stress generally promotes bone formation, whereas tensile and shear stresses may favour resorption or fibrous tissue differentiation [18,19,23]. A more complete mechanical characterisation would require examination of the full stress tensor, including separate principal stress components and hydrostatic–deviatoric decomposition, as employed in advanced mechanoregulation models [24,28]. The choice of von Mises stress in the present study was motivated by its utility as a single intuitive scalar for visual comparison with radiographic observations within a conceptual framework, not by a claim that it fully captures the directional complexity of bone mechanotransduction. Future studies should incorporate directional stress and strain measures to refine the proposed associations.

Despite these limitations, the proposed framework has value because it provides a coherent way to interpret bone structure and bone healing across disciplinary boundaries. It supports the view that stress is not merely an engineering abstraction, but a visible and biologically meaningful determinant of form. Future work should combine quantitative imaging, validated patient-specific finite element modelling, and prospective clinical follow-up to test the associations proposed here and to develop the framework into a clinically applicable decision-support tool.

## 5. Conclusions

This study proposed mechanical stress as a common integrative measure that connects biological adaptation with engineering mechanics in bone. Three principal observations emerged from the conceptual analysis and illustrative finite element visualisation. First, local stress distribution was associated with asymmetric callus formation during fracture healing, suggesting that early repair tissue develops preferentially on the mechanically dominant side of the stress field. Second, stress concentration near geometric discontinuities corresponded to regions of active bone remodelling, supporting the interpretation that bone form reflects the accumulated internal stress balance. Third, non-union under shear-dominant loading conditions was interpreted as a mechanically decompensated state in which the local stress environment did not support stable bridging.

Unlike established frameworks such as Wolff’s law [21], the mechanostat model [22], or computational mechanoregulation [24,25,26], the present approach operates at the clinical–conceptual level, offering a shared observational language for clinicians and engineers without requiring patient-specific computational models. However, stress is positioned as one component within a multifactorial system that includes vascular, cellular, and metabolic contributors to healing. Future validation through region-based mapping of callus distribution against FEM-predicted stress fields, prospective cohort studies, and integration with quantitative imaging would be necessary to transform this conceptual framework into a clinically applicable tool.

## Data Availability

The data presented in this study are contained within the article. The radiographic images presented are anonymised clinical illustrations used solely for conceptual demonstration.
